# A taxonomic account of the genus *Stenodynerus* from China, with descriptions of five new species (Hymenoptera, Vespidae, Eumeninae)

**DOI:** 10.3897/zookeys.595.7734

**Published:** 2016-06-02

**Authors:** Zhen-xia Ma, Bin Chen, Ting-jing Li

**Affiliations:** 1 Institute of Entomology & Molecular Biology, College of Life Sciences, Chongqing Normal University, Chongqing 401331, China

**Keywords:** Hymenoptera, Vespidae, Eumeninae, Stenodynerus, new species, China, distribution

## Abstract

In this paper, 20 species of the genus *Stenodynerus* are reviewed and identified from China, including five new species: *Stenodynerus ninglangensis* Ma & Li, **sp. n.**, *Stenodynerus reflexus* Ma & Li, **sp. n.**, *Stenodynerus similibaronii* Ma & Li, **sp. n.**, *Stenodynerus strigatus* Ma & Li, **sp. n.**, and *Stenodynerus tenuilamellatus* Ma & Li, **sp. n.**, and five new records: *Stenodynerus baronii* Giordani Soika, *Stenodynerus bluethgeni* van der Vecht, *Stenodynerus picticrus* (Thomson), *Stenodynerus pullus* Gusenleitner and *Stenodynerus nepalensis* Giordani Soika. The five new species are described and illustrated in detail. Moreover, the diagnostic characters of all new records and known species from China are provided, with a key to the Chinese species of *Stenodynerus*.

## Introduction

The genus *Stenodynerus* of potter wasps was established by de Saussure (1863). This genus includes 161 species with 26 subspecies worldwide, and is distributed in the Nearctic, Neotropic, Palearctic and Oriental regions. These known species were described or revised by Bohart (1943, 1944, 1948, 1949, 1966, 1980), Buck (2008), Gusenleitner (1981, 1985, 2004, 2008, 2001a, 2001b, 2012), Giordani Soika (1972, 1975, 1976a, 1976b, 1979, 1981, 1985, 1986, 1994), Kim and Yamane (1999, 2004), and so on. So far, ten species with two subspecies have been recorded from China (de Saussure 1863, 1867; André 1884; Morawitz 1889; Giordani Soika 1976; Kurzenko 1977; Giordani Soika 1979; Li 1985; Gusenleitner 2003, 2012; Kim and Yamane 2004). In this study, a total of 20 species of *Stenodynerus* is recognized, including five new species and five newly recorded species. These five new species are described and illustrated in detail, and the diagnostic characters of new records and all known species from China provided. In addition, a key to the Chinese species of *Stenodynerus* is updated. The diagnostic characters and the key were produced based on specimen examination and the information extracted from the literature.

## Materials and methods

Descriptions were made under a stereomicroscope (Olympus SZ61). Measurements were taken as the maximal length of body parts under an image analyzer (LY-M-Tupuwiew), and all figures were taken with a stereomicroscope (LY-WN-YH) attached to a computer. The ratios used throughout the descriptions were measured in the same magnification of the stereomicroscope. Body length was measured from the anterior margin of head to the posterior margin of metasomal tergum II. For the density description of punctures, “sparsely” means that the interspaces are larger than one puncture diameter, “moderately” means equal to the diameter, and “densely” means the interspaces are less than one puncture diameter. Terminology principally follows Carpenter (1982) and Kim and Yamane (2004). Specimens examined are deposited in the Institute of Entomology and Molecular Biology, Chongqing Normal University, Chongqing, China (CQNU).

## Taxonomy

### Stenodynerus

Taxon classificationAnimaliaHymenopteraVespidae

de Saussure, 1863

Stenodynerus de Saussure, 1863: 228; Gusenleitner 1981: 221; Giordani Soika 1994: 133.
Nannodynerus Blüthgen, 1938 (1937): 281.
Parhypodynerus Giordani Soika, 1973: 110.

#### Type species.

*Odynerus chinensis* de Saussure, 1863, designated by Bohart, 1939.

#### Diagnosis.

Body generally small and slender (Figs 1, 7, 14–15, 23, 30, 37, 63, 73); anterior surface of pronotum usually with a pair of median foveae, which sometimes contiguously forms U-shaped (Fig. 69), V-shaped (Figs 3, 18, 40, 48, 52, 57, 60, 65, 75) or a transverse fovea; tegula campanulate, broadest in the middle, length somewhat more than width; parategula just reaching apex of tegula; tergum I generally without a basal transverse carina (Figs 5, 10, 26, 33, 50, 59, 71, 78), but in some Nearctic species present; tergum II without an acarinarium; the terminal segment of male antenna bent backward like a hook, apex usually reaching the base or middle of the segment XI (Fig. 19). This genus is similar in some characters to *Parancistrocerus* Bequaert, which can be distinguished by the presence of an acarinarium on the metasomal tergum II in *Parancistrocerus*.

**Figures 1–6.**
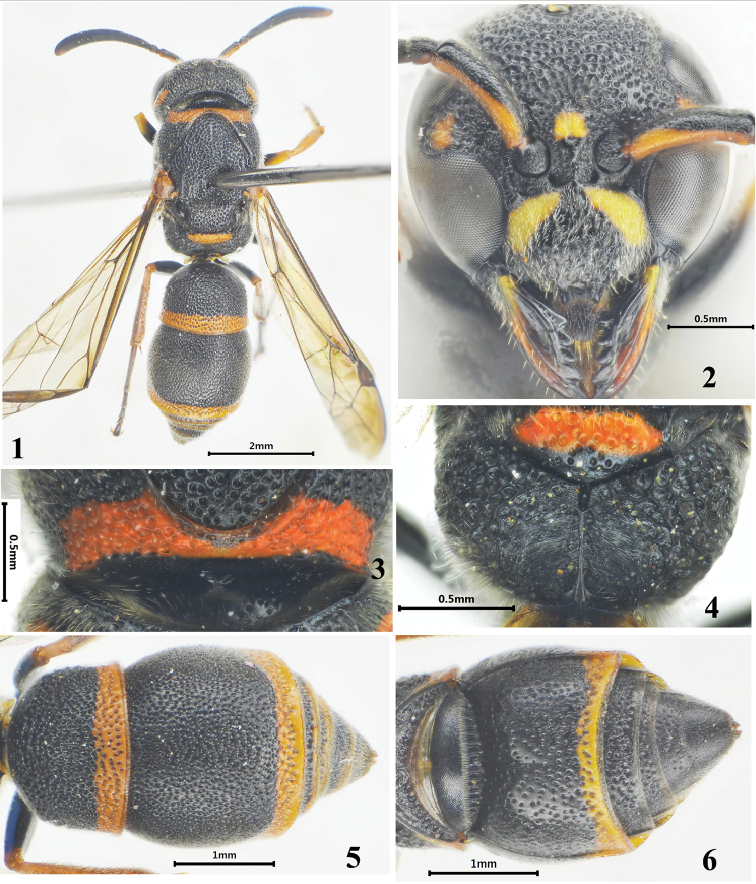
Female of *Stenodynerus ninglangensis* Ma & Li, sp. n. **1** habitus of holotype (dorsal view) **2** clypeus **3** anterior surface of pronotum **4** metanotum and propodeum **5** metasoma (dorsal view) **6** metasomal (ventral view).

**Figures 7–13.**
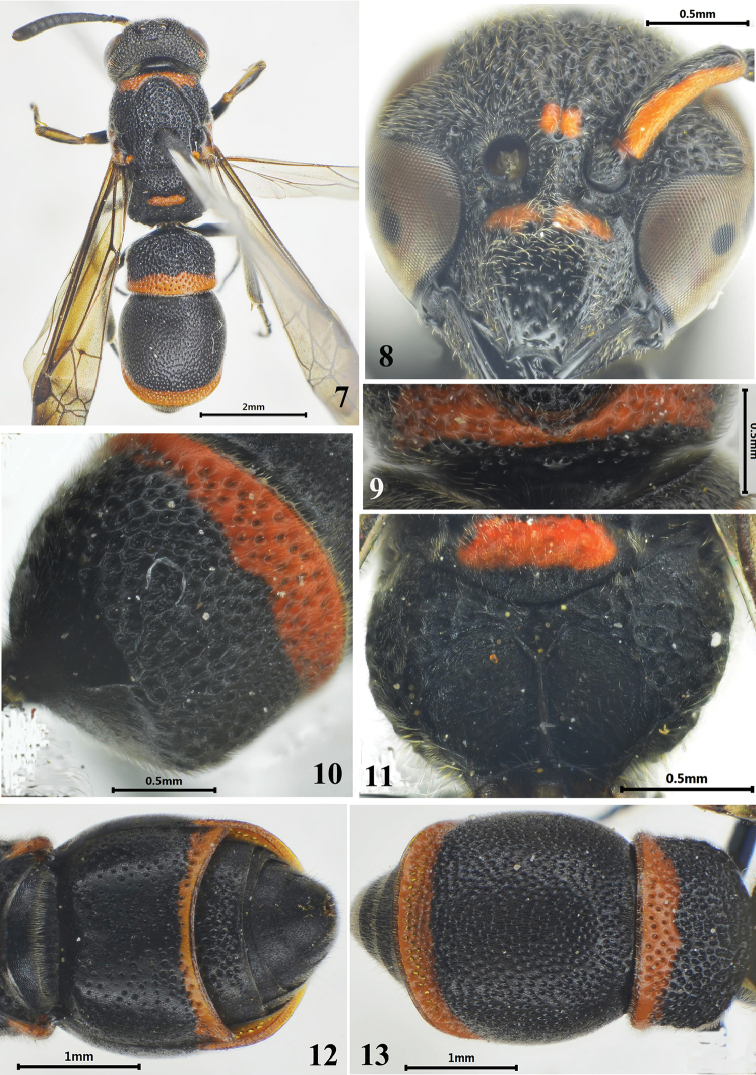
Female of *Stenodynerus reflexus* Ma & Li, sp. n. **7** habitus of holotype (dorsal view) **8** clypeus **9** anterior surface of pronotum **10** metasomal tergum I (dorsal view) **11** metanotum and propodeum **12** metasomal (ventral view) **13** metasoma (dorsal view).

**Figures 14–22.**
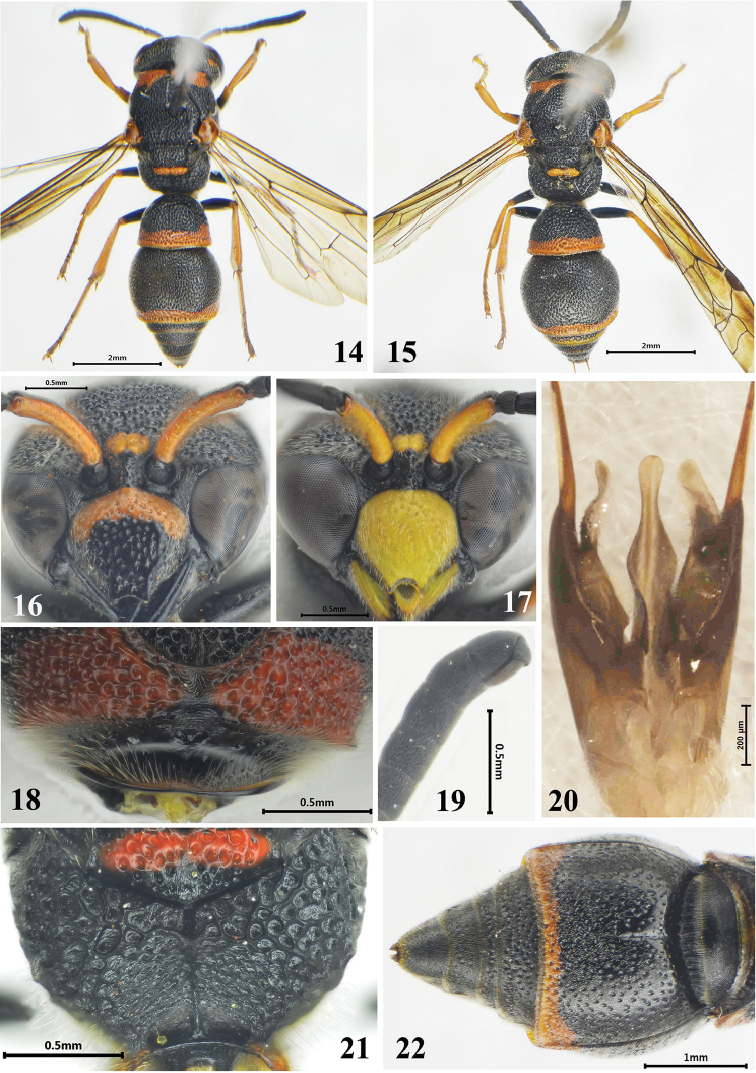
*Stenodynerus similibaronii* Ma & Li, sp. n. **14, 16, 18, 21-22** female, **15, 17, 19–20** male. **14** habitus of holotype (dorsal view) **15** habitus of paratype (dorsal view) **16** clypeus **17** clypeus **18** anterior surface of pronotum **19** antennal segment (lateral view) **20** genitalia (ventral view) **21** metanotum and propodeum **22** metasoma (ventral view).

**Figures 23–29.**
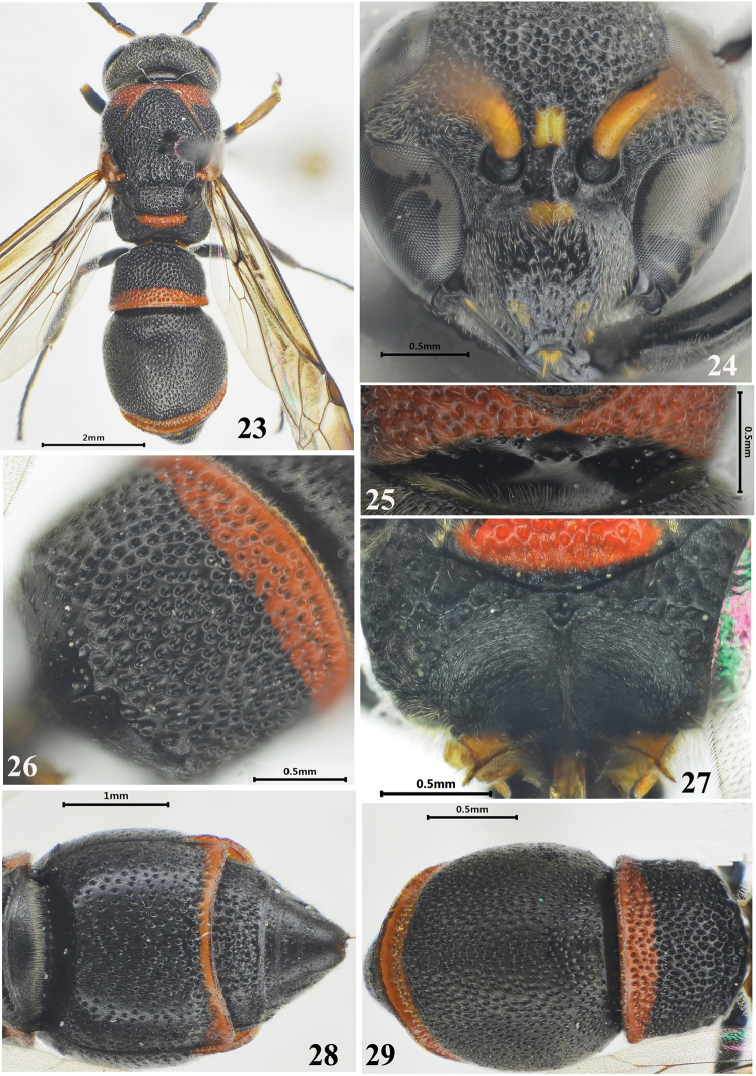
Female of *Stenodynerus strigatus* Ma & Li, sp. n. **23** habitus of holotype (dorsal view) **24** clypeus **25** anterior surface of pronotum **26** metasomal tergum I (dorsal view) **27** metanotum and propodeum **28** metasomal (ventral view) **29** metasoma (dorsal view).

**Figures 30–36.**
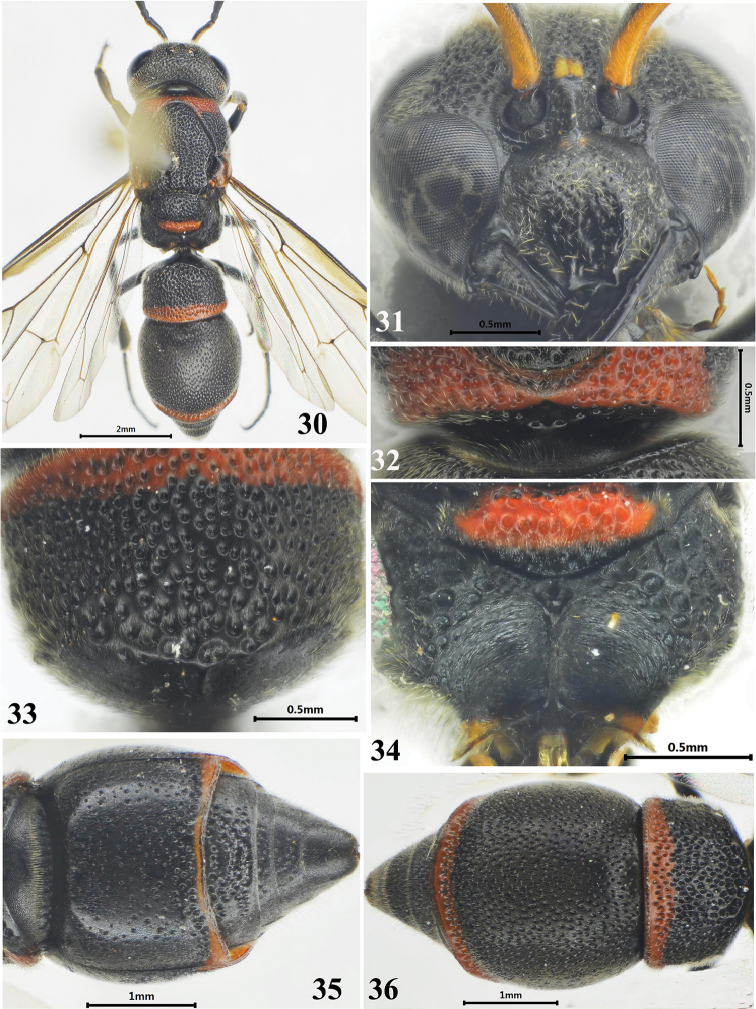
Female of *Stenodynerus tenuilamellatus* Ma & Li, sp. n. **30** habitus of holotype (dorsal view) **31** clypeus **32** anterior surface of pronotum **33** metasomal tergum I (dorsal view) **34** metanotum and propodeum **35** metasomal (ventral view) **36** metasoma (dorsal view).

**Figures 37–45.**
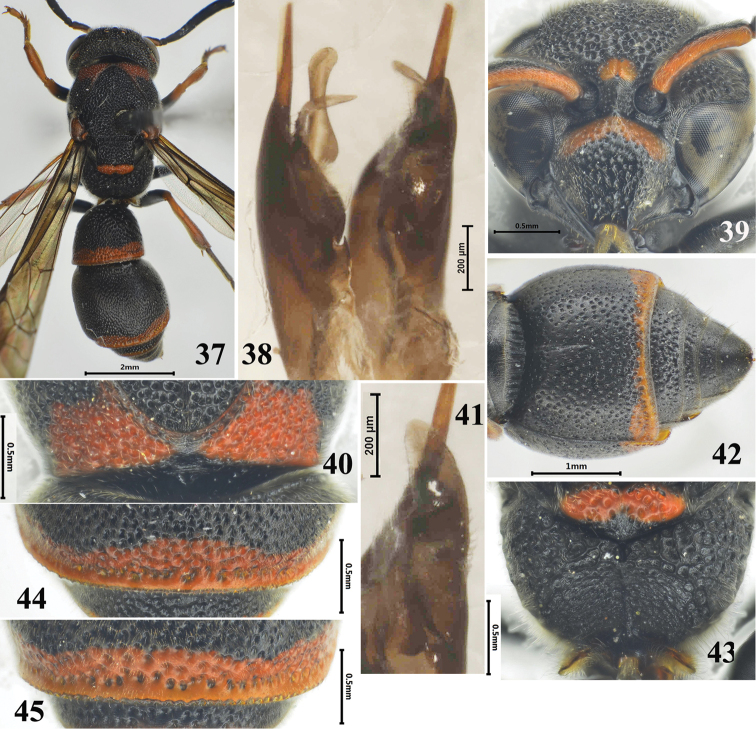
*Stenodynerus baronii*, **45** *Stenodynerus similibaronii*. **37, 39–40, 42–45** female, **38, 41** male **37** habitus (dorsal view) **38** genitalia (ventral view) **39** clypeus **40** anterior surface of pronotum **41** volsella (ventral view) **42** metasomal (ventral view) **43** metanotum and propodeum **44** apex of metasomal tergum II **45** apex of metasomal tergum II.

**Figures 46–50.**
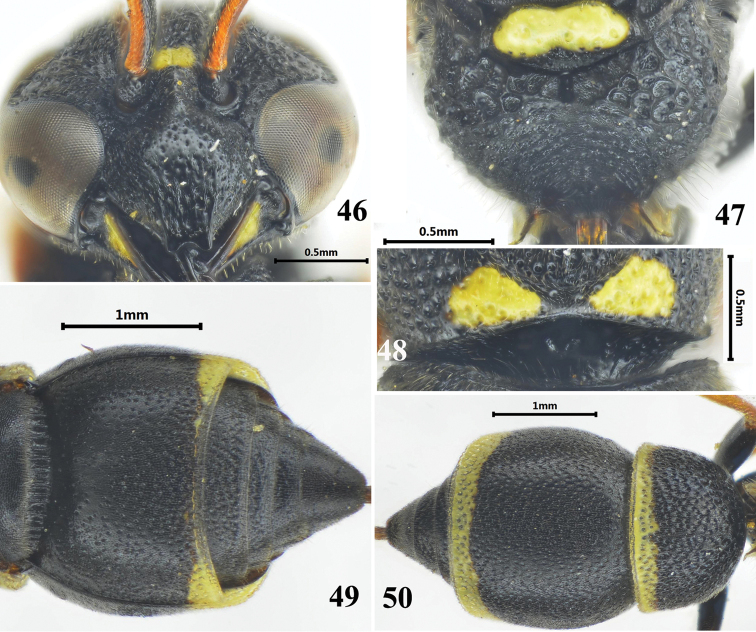
Female of *Stenodynerus bluethgeni*. **46** clypeus **47** metanotum and propodeum **48** anterior surface of pronotum **49** metasomal (ventral view) **50** metasomal (dorsal view).

**Figures 51–55.**
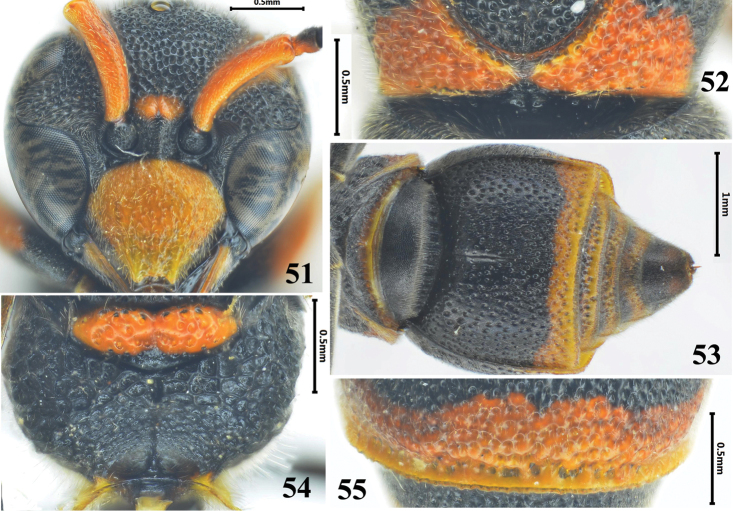
Female of *Stenodynerus nepalensis*. **51** clypeus **52** anterior surface of pronotum **53** metasomal (ventral view) **54** metanotum and propodeum **55** apex of metasomal tergum II.

**Figures 56–59.**
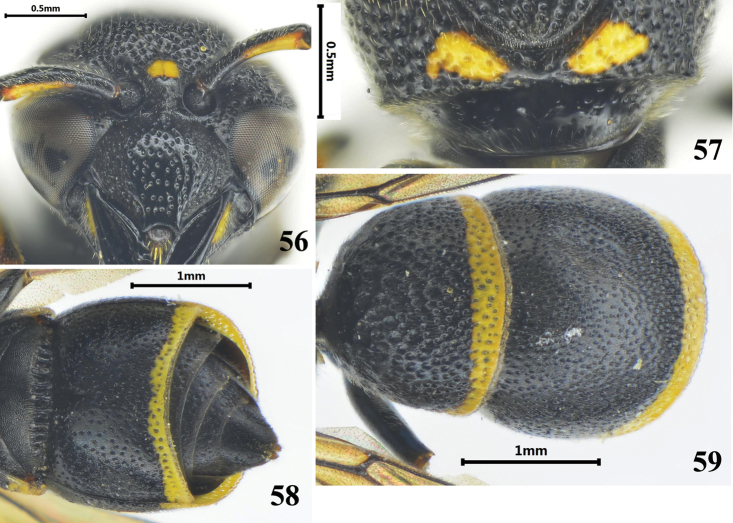
Female of *Stenodynerus pullus*. **56** clypeus **57** anterior surface of pronotum **58** metasomal (ventral view) **59** metasomal (dorsal view).

**Figures 60–62.**
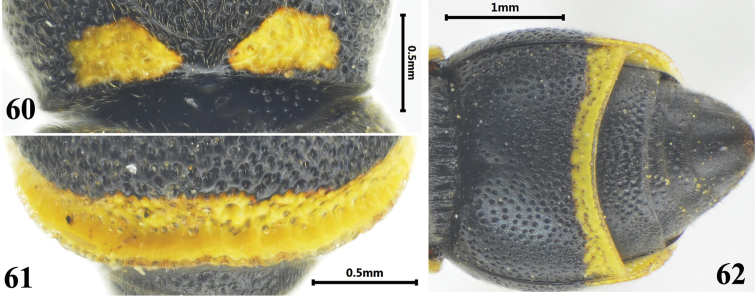
Female of *Stenodynerus tergitus*. **60** anterior surface of pronotum **61** apex of metasomal tergum II **62** metasomal (ventral view).

**Figures 63–68.**
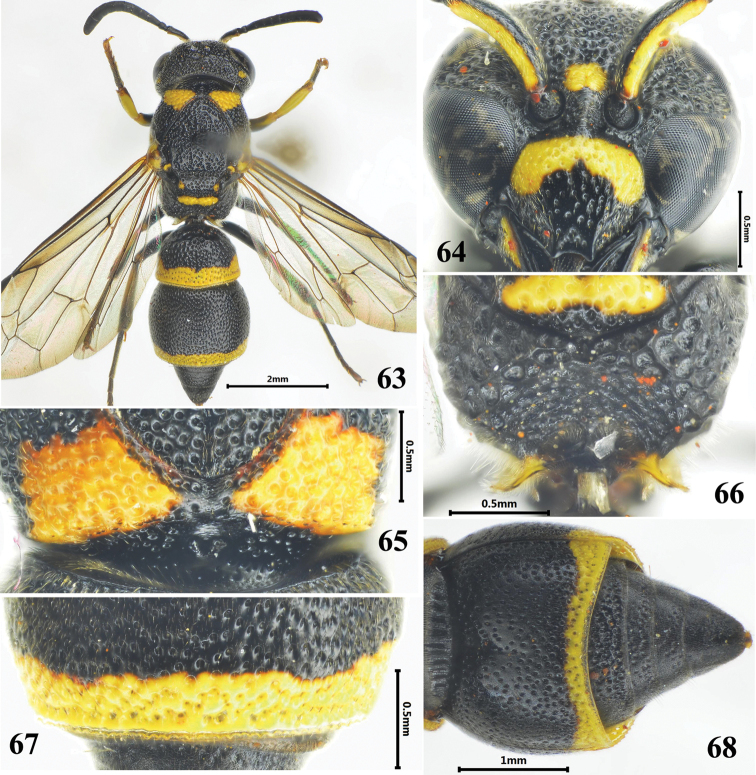
Female of *Stenodynerus chinensis chinensis*. **63** habitus (dorsal view) **64** clypeus **65** anterior surface of pronotum **66** metanotum and propodeum **67** apex of metasomal tergum II **68** metasomal (ventral view).

**Figures 69–72.**
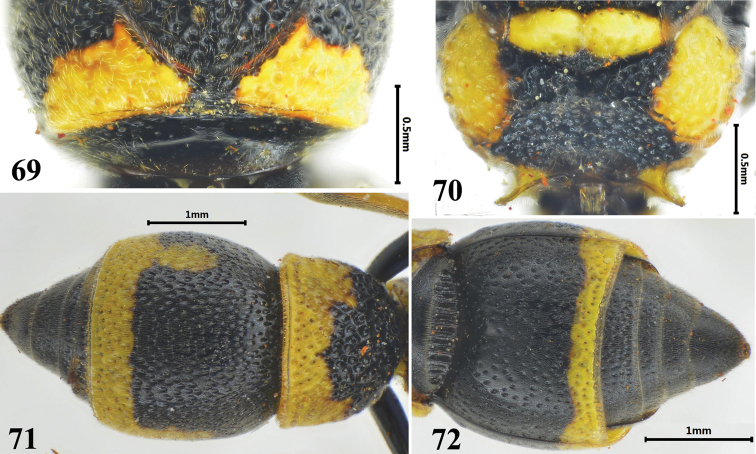
Female of *Stenodynerus frauenfeldi*. **69** anterior surface of pronotum **70** metanotum and propodeum **71** metasomal (dorsal view) **72** metasomal (ventral view).

**Figures 73–76.**
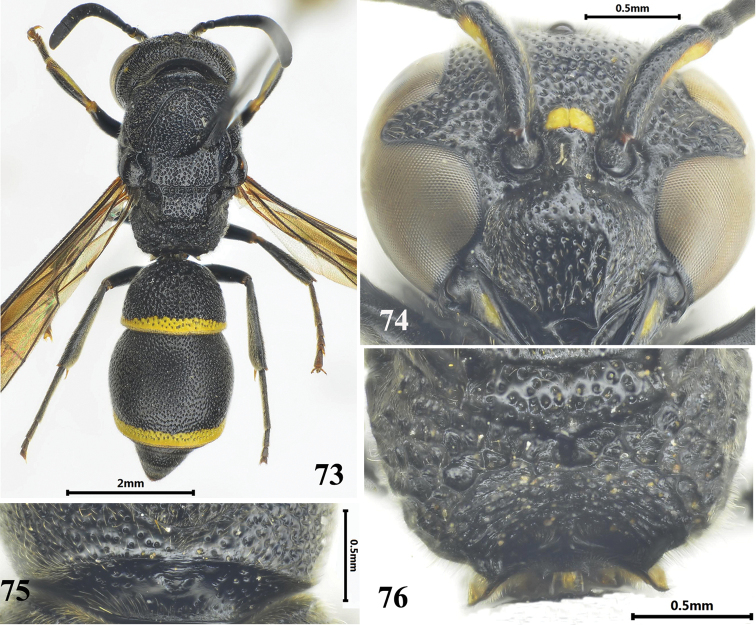
Female of *Stenodynerus funebris*. **73** habitus (dorsal view) **74** clypeus **75** anterior surface of pronotum **76** metanotum and propodeum.

**Figures 77–82.**
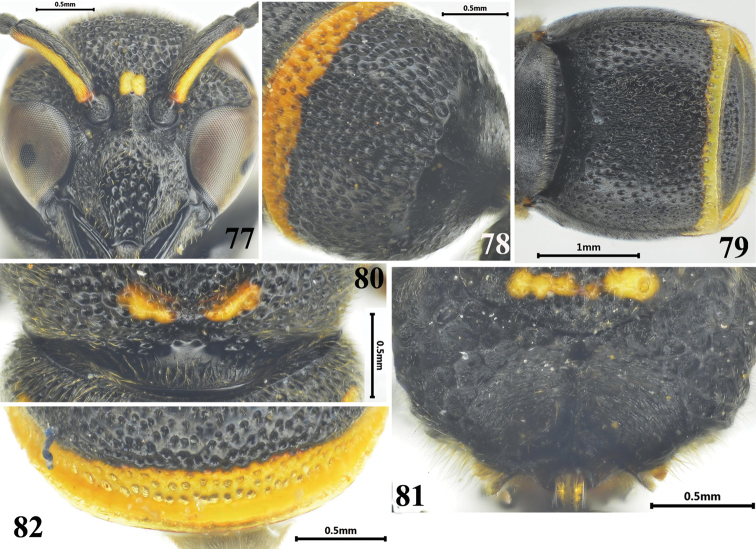
Female of *Stenodynerus pappi pappi*. **77** clypeus **78** metasomal tergum I (dorsal view) **79** metasomal (ventral view) **80** anterior surface of pronotum **81** metanotum and propodeum **82** apex of metasomal tergum II.

#### Distribution.

Nearctic, Neotropic, Palearctic and Oriental regions.

### Stenodynerus ninglangensis

Taxon classificationAnimaliaHymenopteraVespidae

Ma & Li sp. n.

http://zoobank.org/E2130F59-2C6F-494A-891D-A0E2597FFFCD

Figs 1–6

#### Material examined.

Holotype, ♀, China, Yunnan Prov., Lijiang City, Ninglang County, Daxing Town, 27°16'37.68"N, 100°51'03.11"E, 2252 m, 26.VII. 2011, Tingjing Li, No. 1004068 (CQNU). Paratype: 1♀, same data as holotype, No. 1004069 (CQNU).

#### Description.

Female (Figs 1–6): body length 7.2 mm, forewing length 5.6 mm. Black; a basal band on clypeus except median interruption (Fig. 2) and inter-antennal spot yellow; the outer margin of mandible yellow to brown; the spots pale ferruginous: ventral scape, apex of ocular sinus (Fig. 2), transverse post-ocular spot, a band on dorsal surface of pronotum, mesepisternal spot, parategula, metanotum except posterior apex ventrally, apexes of femora, all tibiae and tarsi, and apical bands on metasomal terga I–II and sternum II; tegula brown.

Head. Clypeus strongly punctate, with dense setae, its width much more than length (width 1.25 × length), apex emarginated, apical width: emargination depth = 0.20: 0.04, total width: apical width = 0.86: 0.20 (Fig. 2); frons densely punctate, punctures on vertex somewhat sparser than those on frons; frons and vertex with very sparse and short setae; cephalic fovea obsolete.

Mesosoma. Pronotum, mesoscutum and scutellum densely punctate and reticulate, punctures somewhat larger and sparser than those on the head; punctures on metanotum sparser than those on other parts of the mesosoma. Anterior surface of pronotum shining, almost vertical, with few minute punctures, median foveae contiguous and V-shaped, pronotal carina complete (Fig. 3); scutellum distinct convex; metanotum oblique; dorsal and lateral surfaces of propodeum reticulate-punctate, dorsal surface broad with a weak shelf, posterior surface concave with long and transverse rugae and a median longitudinal carina (Fig. 4).

Metasoma. In dorsal view, tergum I domed, densely punctate, width 1.31 ×length and 0.85 × width of tergum II; tergum II with smaller and sparser punctures than tergum I, apex with deeper and denser punctures than other parts of tergum II, and apical margin without a distinct lamella, (Fig. 5); sternum II sparsely punctate, with a long median longitudinal furrow basally, and its anterior surface sloping (Fig. 6).

Male. Unknown.

#### Remarks.

This species is easily distinguished from all the other members of *Stenodynerus* by the following character combinations: a basal band on clypeus except median interruption yellow (Fig. 2), apex of ocular sinus ferruginous (Fig. 2), apical margin of tergum II without a distinct lamella (Fig. 5), sternum II with a long median longitudinal furrow basally, and its anterior surface sloping (Fig. 6).

#### Distribution.

China (Yunnan).

#### Etymology.

The specific name *ninglangensis* is the Latin adjective of the region from which the type specimens were collected.

### Stenodynerus reflexus

Taxon classificationAnimaliaHymenopteraVespidae

Ma & Li sp. n.

http://zoobank.org/18D6BDFC-DD54-4DAA-8B2D-38E7B0670C63

Figs 7–13

#### Material examined.

Holotype, ♀, China, Yunnan Prov., Baoshan City, Tengchong County, Beihai Village, 25°16'48.82"N, 98°34'49.16"E, 1783 m, 13.VII.2011, Xin Zhou, No. 1004070 (CQNU).

#### Description.

Female (Figs 7–13): body length 7.4 mm, forewing length 5.8 mm. Black; with the following spots ferruginous: clypeus except median interruption basally (Fig. 8), inter-antennal spot, ventral scape, post-ocular spot, a broad band on the pronotal dorsum, mesepisternal spot, parategula, metanotum except posterior apex ventrally, apical bands on metasomal terga I–II and sternum II, a long band on the dorsal surface of the fore tibia; tegula brown.

Head. Clypeus with sparse punctures, and with setae basally and laterally, its width much more than length (width 1.24 × length), apex moderately emarginated, apical width: emargination depth = 0.25: 0.08, total width: apical width = 0.88: 0.25 (Fig. 8); frons densely punctate, punctures on vertex somewhat weaker than those on frons; frons and vertex with dense setae; cephalic fovea obsolete.

Mesosoma. Masosoma densely punctate and reticulate except metanotum, punctures generally larger than those on the head; punctures on metanotum sparser and shallower than those on other parts of the mesosoma. Anterior surface of pronotum slightly sloping, distinctly punctate, and with a pair of round separated median foveae, the interspace between these two median foveae less than one fovea diameter, pronotal carina complete (Fig. 9); scutellum distinctly convex; metanotum nearly vertical; dorsal and lateral surface of propodeum reticulate-punctate, dorsal surface narrow with a developed shelf, posterior surface concave with long and transverse rugae and a median longitudinal carina (Fig. 11).

Metasoma. In dorsal view, tergum I campanulate, coarsely punctate, width 1.37 × length and 0.85 × width of tergum II, anterior surface vertical, almost impunctate, and with a median longitudinal carina in upper half (Fig. 10); tergum II with smaller and sparser punctures than tergum I, and with a broad reflex apical lamella, dense and deep punctures forming a wide transverse groove on the base of lamella (Fig. 13); sternum II sparsely punctate, basally with a very short median longitudinal furrow, and its anterior surface almost vertical (Fig. 12).

Male. Unknown.

#### Remarks.

This species is similar to *Stenodynerus pappi* by having the propodeal shelf developed, a median longitudinal carina on propodeal concavity (Fig. 11, 81), anterior vertical surface of tergum I with a longitudinal median carina in upper half (Fig. 10). It is different from *Stenodynerus pappi* and other members of the genus in the following characters: clypeus basally ferruginous except median interruption (Fig. 8), a broad band on pronotum (Fig. 9), and sternum II with a very short median longitudinal furrow basally, and its anterior surface almost vertical (Fig. 12).

#### Distribution.

China (Yunnan).

#### Etymology.

The specific name is the Latin adjective *reflexus*, which refers to the apical lamella of metasomal tergum II broadly reflexed.

### Stenodynerus similibaronii

Taxon classificationAnimaliaHymenopteraVespidae

Ma & Li sp. n.

http://zoobank.org/3B9079BB-25FC-4436-8C61-9A2AFE5E7CC1

Figs 14–22 , 45

#### Material examined.

Holotype, ♀, China, Yunnan Prov., Baoshan City, Tengchong County, Shangying Village, 25°0'54.72"N, 98°39'12.86"E, 1823 m, 31.VII.2015, Zhenxia Ma & Long Li, No. 1004071 (CQNU). Paratypes: 1♀♀2♂♂, the same data as holotype, Nos. 1004072, 10040743, 1004074 (CQNU).

#### Description.

Female (Figs 14, 16, 18, 21–22, 45): body length 7.0 mm, forewing length 6.5 mm. Black; with the following spots pale ferruginous: a basal band of clypeus (Fig. 16), ventral scape, inter-antennal spot, post-ocular spot, spots on outsides of tegula anteriorly and posteriorly, parategula, apexes of femora to terminal tarsi, and apical bands on metasomal terga I–II and sternum II; a band on dorsal surface of pronotum except median interruption (Fig. 18), and metanotum except posterior apex ventrally dark ferruginous (Fig. 21); tegula brown.

Head. Clypeus with moderate punctures, lateral surface with sparse setae, its width somewhat more than length (width 1.09 × length), apex slightly emarginated, apical width: emargination depth = 0.33: 0.07, total width: apical width = 1.00: 0.33 (Fig. 16); frons and vertex densely punctate and reticulate; frons with sparse and very short setae, setae on vertex denser than those on frons.

Mesosoma. Masosoma densely punctate and reticulate; punctures generally larger than those on the head; punctures on pronotal dorsum and mesoscutum somewhat denser than those on other parts of the masosoma (Fig. 14). Anterior surface of pronotum almost vertical with few small punctures, median foveae contiguous and V-shaped, a few short transverse carinae above median foveae, pronotal carina interrupted medially (Fig. 18); scutellum distinctly convex; metanotum oblique; dorsal and lateral surfaces of propodeum reticulate-punctate, dorsal surface broad with a weak shelf, posterior surface concave with long and transverse rugae and a median longitudinal carina (Fig. 21).

Metasoma. In dorsal view, tergum I domed, densely punctate, width 1.35 × length and 0.84 × width of tergum II; tergum II with smaller and sparser punctures than tergum I, apex with deeper and denser punctures than other parts of tergum II, and apical margin without a distinct lamella (Fig. 45); sternum II sparsely punctate, with a long median longitudinal furrow basally, and its anterior surface sloping (Fig. 22).

Male (Figs 15, 17, 19, 20): body length 6.8 mm, forewing length 5.6 mm. Sculpture, punctuation, setae and coloration similar to those of female except the follows: entire clypeus, mandible except apical portion, ventral scape and inter-antennal spot yellow; clypeus strongly, convex medially, with sparse and small punctures, its width equal to length, apex deeply emarginated and U-shaped, apical width: emargination depth = 0.27: 0.12, total width: apical width = 0.8: 0.27 (Fig. 17); punctures on apex of tergum II deeper than those in female; width of tergum I 1.45 × length and 0.79 × width of tergum II; the terminal segment of antenna bent backward like a hook, apex reaching the base of segment XI (Fig. 19). Male genitalia as in Fig. 20, volsella with setae and slightly truncate apically, parallel spines elongate without setae, penis valve rounded apically.

#### Remarks.

This species is similar to *Stenodynerus baronii* by a basal band of clypeus (Fig. 16), metasomal sternum II with a long median longitudinal furrow basally (Fig. 22), and propodeal concavity with a median longitudinal carina (Fig. 21). It is different from *Stenodynerus baronii* and other members of the genus in the following characters: anterior surface of pronotum with wider V-shaped median foveae (Fig. 18), punctures on apex of metasomal tergum II sparser (Fig. 45), and male volsella of genitalia narrower and slightly truncate apically than the corresponding parts in *Stenodynerus baronii* (Figs 20, 41).

#### Distribution.

China (Yunnan).

#### Etymology.

The specific name *similibaronii* is a Latin adjective which refers to the similar species of *Stenodynerus baronii*.

### Stenodynerus strigatus

Taxon classificationAnimaliaHymenopteraVespidae

Ma & Li sp. n.

http://zoobank.org/45B4C174-8CAA-4972-8642-91F292686599

Figs 23–29

#### Material examined.

Holotype, ♀, China, Shaanxi Prov., Ankang City, Langao County, Huanli Town, 32°12'52.77"N, 109°0'17.90"E, 1808 m, 7.VIII.2015, Yan Peng & Wenkai Zhou, No. 1004075 (CQNU).

#### Description.

Female (Figs 23–29): body length 8.3 mm, forewing length 7.7 mm. Black; the following spots yellow: a basal transverse median spot and two small obscure apical spots on clypeus (Fig. 24), inter-antennal spot, and scape ventrally; small post-ocular spot, pronotal dorsum except posterior apex, mesepisternal spot, parategula, metanotum largely (Fig. 27), apical bands on metasomal terga I–II and sternum II, and a long band on fore and mid tibiae dorsally ferruginous; tegula brown.

Head. Clypeus convex medially, moderately punctate, somewhat reticulate, with sparse and short setae, its width more than length (width 1.25 × length), apex slightly emarginated, apical width: emargination depth = 0.29: 0.06, total width: apical width = 1.02: 0.29 (Fig. 24); frons and vertex densely punctate, with short setae; cephalic fovea obsolete.

Mesosoma. Masosoma densely punctate and reticulate, punctures generally larger than those on the head; punctures on pronotal dorsum denser than those on others parts of the masosoma (Fig. 23). Anterior surface of pronotum sloping, shinning, with few punctures and a pair of round separated median foveae, the interspace between these two median foveae much more than one diameter, pronotal carina complete (Fig. 25); scutellum distinctly convex; metanotum nearly vertical; dorsal and lateral surfaces of propodeum reticulate-punctate, dorsal surface narrow with a weak shelf, posterior surface concave with long and transverse rugae and a median longitudinal carina (Fig. 27).

Metasoma. In dorsal view, tergum I campanulate, coarsely punctate, width 1.58 × length and 0.81 × width of tergum II, anterior surface vertical, almost impunctate, and with a median longitudinal carina and two transverse striations (Fig. 26); tergum II with smaller and sparser punctures than tergum I, and with a broad reflex apical lamella, dense and deep punctures forming a wide transverse groove on the base of lamella (Fig. 29); sternum II sparsely punctate, without a median longitudinal furrow basally, and its anterior surface almost vertical (Fig. 28).

Male. Unknown.

#### Remarks.

This species is similar to *Stenodynerus pappi* by a median longitudinal carina on propodeal concavity (Fig. 27), anterior vertical surface of tergum I with a longitudinal median carina in upper half (Fig. 26), and tergum II with a broad reflex apical lamella (Fig. 29). It is different from *Stenodynerus pappi* and other members of the genus in the following characters: a transverse median spot and two obscure apical spots on clypeus basally yellow (Fig. 24), pronotal dorsum mostly ferruginous (Fig. 25), the interspace of pronotal median foveae much more than one fovea diameter (Fig. 25), anterior vertical surface of tergum I with two transverse striations (Fig. 26), and sternum II without a median longitudinal furrow basally, and its anterior surface almost vertical (Fig. 28).

#### Distribution.

China (Shaanxi).

#### Etymology.

The specific name is the Latin adjective *strigatus*, which refers to the anterior vertical surface of tergum I with two transverse striations.

### Stenodynerus tenuilamellatus

Taxon classificationAnimaliaHymenopteraVespidae

Ma & Li sp. n.

http://zoobank.org/3691DE51-0508-4583-913B-C873FC5E66AD

Figs 30–36

#### Material examined.

Holotype, ♀, China, Yunnan Prov., Baoshan City, Tengchong County, Zhonghe Village, 25°31'55.10"N, 98°23'44.21"E, 1663 m, 29.VII.2015, Zenghui Huang & Siyu Xie, No. 1004076 (CQNU). Paratype: 1♀, China, Yunnan Prov., Baoshan City, Tengchong County, Jietou Village, 25°25'11.18"N, 98°39'42.75"E, 1631 m, 15.VII.2006, Li Ma, No. 1004077 (CQNU).

#### Description.

Female (Figs 30–36): body length 8.0 mm, forewing length 6.7 mm. Black; a minute spot on clypeus basally, inter-antennal spot, and scape ventrally yellow; with the following parategula, metanotum except posterior apex ventrally, apical bands on metasomal terga I–II and sternum II, and the dorsal surface of fore femur; tegula brown.

Head. Clypeus convex medially with sparse punctures and setae, its width somewhat more than length (width 1.08 × length), apex slightly emarginated, apical width: emargination depth = 0.26: 0.07, total width: apical width = 0.96: 0.26 (Fig. 31); frons and vertex densely punctate and reticulate, with short setae; cephalic fovea obsolete.

Mesosoma. Masosoma densely punctate and reticulate, punctures generally larger than those on the head; punctures on pronotal dorsum denser than those on other parts of the masosoma (Fig. 30). Anterior surface of pronotum somewhat sloping, with a few punctures and a pair of round separated median foveae, the interspace between two median foveae almost equal to one fovea diameter, pronotal carina complete (Fig. 32); scutellum distinctly convex; metanotum nearly vertical; dorsal and lateral surfaces of propodeum reticulate-punctate; dorsal surface narrow with a moderate shelf; posterior surface concave with long and transverse rugae and a median longitudinal carina (Fig. 34).

Metasoma. In dorsal view, tergum I campanulate, coarsely punctate, width 1.59 × length and 0.81 × width of tergum II, anterior surface vertical, almost impunctate, and with a median longitudinal carina in upper half (Fig. 33); tergum II with smaller and sparser punctures than tergum I, and with a narrow reflex apical lamella, a row of deep and dense punctures forming a narrow transverse groove on the base of lamella (Fig. 36); sternum II sparsely punctate, without a median longitudinal furrow basally, and its anterior surface vertical (Fig. 35).

Male. Unknown.

#### Remark.

This species is similar to *Stenodynerus pappi* by a median longitudinal carina on propodeal concavity (Fig. 34), and anterior vertical surface of tergum I with a longitudinal median carina in upper half (Fig. 33). It is different from *Stenodynerus pappi* and other members of the genus in the following characters: clypeus with a minute spot basally (Fig. 31); propodeal shelf moderately (Fig. 34); apical lamella of metasomal tergum II distinctly narrower than that of *Stenodynerus pappi* (Figs 36, 82), and sternum II without a median longitudinal furrow basally, and its anterior surface vertical (Fig. 35).

#### Distribution.

China (Yunnan).

#### Etymology.

The specific name *tenuilamellatus* is derived from two Latin words: *tenuis* (= narrow) and *lamellatus* (= lamella), which refers to metasomal tergum II with a narrow apical lamella.

### Stenodynerus baronii

Taxon classificationAnimaliaHymenopteraVespidae

Giordani Soika, 1975 new record

Figs 37–44

Stenodynerus baronii Giordani Soika, 1975: 387; 1994: 133, 137.

#### Material examined.

1♀2♂♂, China, Tibet Autonomous Region, Nyingchi City, Medog County, Medog Town, 27.VII.2014, Tingjing Li.

#### Diagnosis.

A basal band of clypeus ferruginous (Fig. 39), with moderate punctures, its width more than length; anterior surface of pronotum almost vertical, with few small punctures, median foveae small and V-shaped, a few short transverse carinae above median foveae, pronotal carina interrupted medially (Fig. 40); propodeal shelf almost obsolete, propodeal concavity with a long median longitudinal carina (Fig. 43); punctures on apex of metasomal tergum II strongly dense, deep and irregular (Fig. 44); and sternum II with a long median longitudinal furrow basally (Fig. 42); male genitalia as in Fig. 38, volsella with rounded apically, penis valve rounded apically.

#### Distribution.

China (new record: Tibet), Bhutan.

### Stenodynerus bluethgeni

Taxon classificationAnimaliaHymenopteraVespidae

van der Vecht, 1971 new record

Figs 46–50

Odynerus pictus Herrich-Schaeffer, 1839: 13, 32.
Odynerus dentisquama : Morawitz 1895: 462.
Euodynerus dentisquama : Blüthgen 1938 (1937): 281.
Nannodynerus dentisquama : Blüthgen 1952: 2; 1961: 107, 111; Blüthgen and Königsmann 1969: 928.
Stenodynerus dentisquama : Giordani Soika 1970: 110.
Stenodynerus bluethgeni van der Vecht, 1971: 131; van der Vecht and Fischer 1972: 65; Gusenleitner 1981: 217, 221.

#### Material examined.

3♀♀1♂, China, Shaanxi Prov., Ankang City, Jianming Town, 16.VIII.2012, Xin Zhou & Cheng Yang; 1♀, China, Jilin Prov., Yanji City, Xiaoying Town, Mingzhu Village, 3.VII.2012, Ju You & Yuan Bai.

#### Diagnosis.

Body with pale yellow spots (Figs 46–50). Cephalic fovea distinct, width less than the distance between posterior ocelli; clypeus black, medially convex (Fig. 46); anterior surface of pronotum almost vertical, with a few small punctures, median foveae V-shaped, pronotal carina complete (Fig. 48); propodeal shelf absent, propodeal concavity with a very short median longitudinal carina (Fig. 47); metasomal tergum II with very small and shallow punctures, without a distinct apical lamella (Fig. 50); sternum II black except lateral surface, without a median longitudinal furrow basally, and its anterior surface sloping (Fig. 49).

#### Distribution.

China (new record: Jilin, Shaanxi), Netherlands, Belgium, Germany, France, Spain, Italy, Switzerland, Austria, Hungary, Czechoslovakia, Belarus, Russia, Serbia, Bulgaria, Albania, Greece, Cyprus, Turkey, Iran.

### Stenodynerus nepalensis

Taxon classificationAnimaliaHymenopteraVespidae

Giordani Soika, 1985 new record

Figs 51–55

Stenodynerus nepalensis Giordani Soika, 1985: 37, 40; 1994: 135, 143; Gusenleitner 1987: 255; 2001b: 659.

#### Material examined.

5♀♀5♂♂, China, Yunnan Prov., Diqing Zang Autonomous Prefecture, Deqin County, Fushan Town, 22.VII.2014, Tingjing Li; 3♀♀5♂♂, China, Yunnan Prov., Diqing Zang Autonomous Prefecture, Deqin County, Yunling Town, 21.VII.2014, Tingjing Li.

#### Diagnosis.

Clypeus with strong punctures, width somewhat more than length (Fig. 51); anterior surface of pronotum vertical, with few punctures, median foveae V-shaped, pronotal carina interrupted medially (Fig. 52); propodeal shelf absent (Fig. 54); metasomal tergum II without a distinct apical lamella, punctures on apex deep, dense and irregular (Fig. 55); sternum II with a long median longitudinal furrow basally, and its anterior surface sloping (Fig. 53).

#### Distribution.

China (new record: Yunnan), Nepal, India, Thailand.

### Stenodynerus pullus

Taxon classificationAnimaliaHymenopteraVespidae

Gusenleitner, 1981 new record

Figs 56–59

Stenodynerus pullus Gusenleitner, 1981: 209, 220, 246; Kim 1999: 348, 352; Kim and Yamane 2004: 240, 260–262.

#### Material examined.

2♀♀, China, Inner Mongolia Autonomous Region, Helan Mountain, Gulabenxiaosong Hills, 30.VII.2010, Zejian Li & Junzhe Xue; 1♂, China, Inner Mongolia Autonomous Region, Helan Mountain, Shuimogou, 27.VII.2010, Fangzhou Ma.

#### Diagnosis.

Cephalic fovea obsolete; clypeus black, medially convex, with small punctures (Fig. 56); pronotal dorsum with a pair of spots, anterior surface almost vertical, with a few small punctures, median foveae V-shaped, pronotal carina complete (Fig. 57); propodeum without shelf, propodeal concavity with a very short median longitudinal carina; metasomal tergum II without a distinct apical lamella (Fig. 59); sternum II with a very short median longitudinal furrow basally, and its anterior surface sloping (Fig. 58).

#### Distribution.

China (new record: Inner Mongolia), Turkey, Russia, Mongolia, Korea.

### Stenodynerus tergitus

Taxon classificationAnimaliaHymenopteraVespidae

Kim, 1999 new record

Figs 60–62

Stenodynerus tergitus Kim, 1999: 349, 352; Kim and Yamane 2004: 238-239, 250.

#### Material examined.

1♀3♂♂, China, Shaanxi, Prov., Weinan City, Luonan County, Mantoushan, 8.VIII.2012, Xin Zhou & Cheng Yang.

#### Diagnosis.

Cephalic fovea small and shallow, width less than the distance between posterior ocelli; clypeus black, medially convex, its width somewhat more than length; anterior surface of pronotum sloping, with a few small punctures, median foveae V-shaped, prontoal carina complete (Fig. 60); tergum II with a broad reflex lamella apically, deep and dense punctures forming a broad transverse groove on the base of lamella (Fig. 61); sternum II with a median longitudinal furrow basally, and its anterior surface sloping (Fig. 62).

#### Distribution.

China (new record: Shaanxi), Korea.

### Stenodynerus chinensis chinensis

Taxon classificationAnimaliaHymenopteraVespidae

(de Saussure, 1863)

Figs 63–68

Odynerus chinensis de Saussure, 1863: 230; von Schulthess 1934: 91; van der Vecht 1967: 32; Opinion 1970: 187, 189–191.
Stenodynerus chinensis : van der Vecht and Fischer 1972: 65; Giordani Soika 1972: 105; Gusenleitner 1981: 220, 289; Li 1985: 138; Yamane and Gusenleitner 1996: 43-44.
Stenodynerus chinensis chinensis : Giordani Soika 1986: 124; Kim and Yamane 2004: 239, 256-257.

#### Material examined.

2♀♀, China, Henan Prov., Sanmenxia City, Lushi County, Wulichuan Town, 9.VIII.2012, Ju You & Yuan Bai; 4♀♀, China, Henan Prov., NanYang City, Yuanyang County, Sangping Town, Huangsha Village; 5♀♀1♂, China, Shaanxi Prov., Ankang City, Langao County, Huanli Town, 7.VIII.2015, Zhenxia Ma & Yan Peng; 2♀♀3♂♂, China, Shaanxi Prov., Ankang City, Zhenping County, Zengjia Town, 10.VIII.2015, Zhenxia Ma & Yan Peng; 4♀♀2♂♂, China, Shaanxi Prov., Weinan City, Hua County, Jindui Village 7.VIII.2012, Xin Zhou & Cheng Yang; 3♀♀1♂, China, Shaanxi Prov., Baoji City, Mei County, Huaiya Town, 16.VIII.2015, Zhenxia Ma & Yan Peng; 1♀4♂♂, China, Shannxi Prov., Xian City, Hongqing Town, 19.VIII.2015, Zhenxia Ma & Yan Peng; 7♀♀1♂, China, Shaanxi Prov., Baoji City, Mei County, Jinqu Town, 14.VIII.2015, Zhenxia Ma & Lingquan Zeng; 14♀♀7♂♂, China, Shaanxi Prov., Baoji City, Mei County, Qinghua Town, Jinjiazhuang Village, 15.VIII.2015, Zhenxia Ma & Yan Peng; 8♀♀18♂♂, China, Sichuan Prov., Leshan City, Emeishan County, Huangwan Town, Miaoergang Village, 10.VIII.2011, Tingjing Li; 3♀♀1♂, China, Sichuan Prov., Leshan City, Emeishan County, Longchi Town, Longchi Village, 12.VIII.2011, Tingjing Li & Zhenhu Wu; 3♀♀3♂♂, China, Sichuan Prov., Leshan City, Emeishan County, Zhanggou Village, 8.VIII.2011, Tingjing Li; 12♀♀3♂♂, China, Sichuan Prov., Chongzhou City, Tianshun Village, 17.VIII.2011, Tingjing Li & Zhenhu Wu; 2♀♀1♂, China, Sichuan Prov., Leshan City, Emeishan County, Dawei Town, 13.VIII.2011, Tingjing Li; 4♀♀3♂♂, China, Chongqing Municipality, Youyang County, Banqiao Town, Shuangqiao Village, 26.VIII.2012, Cheng Yang; 6♀♀3♂♂, China, Chongqing Municipality, Jiangjin, Heiwan Village, 23.VI.2012, Xin Zhou; 1♀2♂♂, China, Chongqing Municipality, Liangping County, Bishan Town, Xinyuan Village, 5.IX.2014, Chunfa Chen; 1♀2♂♂, China, Chongqing Municipality, Chengkou County, Bashan Town, Lianmen Village, 7.VIII.2015, Tingjing Li & Chunfa Chen; 1♀2♂♂, China, Chongqing Municipality, Chengkou County, Xianyi Town, Shuangqiao Village, 4.VIII.2015, Tingjing Li & Chunfa Chen; 3♀♀2♂♂, China, Chongqing Municipality, University Town, Xiyongtuanjie, 5.VII.2011, Xin Zhou; 7♀♀11♂♂, China, Chongqing Municipality, Chongqing Normal University, 10.VII.2015, Tingjing Li & Zhenxia Ma; 3♂♂, China, Guizhou Prov., Kaili City, Leishan County, Fangxiang Town, Pingxiang Village, 24.VI.2015, Tingjing Li & Yan Peng.

#### Diagnosis.

Almost whole body covered with large and dense punctures, strongly sculptured (Fig. 63). Cephalic fovea small and shallow, width less than the distance between posterior ocelli; basal half of clypeus yellow, with sparse punctures (Fig. 64); anterior surface of pronotum sloping, with distinct and strong punctures, median foveae V-shaped (Fig. 65); propodeal shelf weak, posterior surface concave, with a median longitudinal carina in lower half (Fig. 66); metasomal tergum II without a distinct apical lamella, apex with deep punctures (Fig. 67); sternum II with a long median longitudinal furrow basally, and its anterior surface sloping (Fig. 68).

#### Distribution.

China (Hebei, Sichuan; Shannxi, Henan, Yunnan, Chongqing, Guizhou, Taiwan).

### Stenodynerus copiosus

Taxon classificationAnimaliaHymenopteraVespidae

Gusenleitner, 2012

Stenodynerus copiosus Gusenleitner, 2012: 1132–1134.

#### Material examined.

No specimens examined.

#### Diagnosis.

Male: clypeus yellow, with small punctures, its width almost equal to length; pronotal carina interrupted medially; metasomal tergum II without a distinct apical lamella; sternum II with a median longitudinal furrow basally; female: unknown (Gusenleitner 2012).

#### Distribution.

China (Shanxi, Shaanxi).

### Stenodynerus frauenfeldi

Taxon classificationAnimaliaHymenopteraVespidae

(de Saussure, 1867)

Figs 69–72

Odynerus frauenfeldi de Saussure, 1867: 15; von Schulthess 1934: 91; Yasumatsu 1935: 225.
Odynerus nigriclypeatus Sonan, 1930: 356; Giordani Soika 1986: 124.
Odynerus apiciornatus : Yano 1932: 309.
Stenodynerus frauenfeldi : van der Vecht and Fischer 1972: 67; Giordani Soika 1972: 105; 1986: 124; 1994: 135, 152; Gusenleitner 1981: 220, 287; Kim 1999: 348, 350; Kim and Yamane 2004: 239, 251–252.

#### Material examined.

1♀2♂♂, China, Jilin Prov., Yanji City, Xiaoying Town, 4.VI.2104, Ju You & Yuan Bai; 2♀♀, China, Jilin Prov., Baishan City, Linjiang County, Naozhi Town, 7.VII.2012, Ju You & Yuan Bai; 3♀♀48♂♂, China, Shannxi Prov., Huayin City, Hua Mountain, 5.VIII.2012, Xin Zhou & Ju You; 5♀♀54♂♂, China, Shannxi Prov., Ankang City, Jianming Town, 15.VIII.2012, Xin Zhou & Cheng Yang; 2♀♀3♂♂, China, Shannxi Prov., Shangluo City, Luonan County, Mantoushan, 8.VIII.2012, Ju You & Yuan Bai; 3♂♂, China, Shannxi Prov., Hanzhong City, Liuba County, Jiangkou Town, 18.VIII.2012, Xin Zhou & Cheng Yang; 42♀♀11♂♂, China, Shanxi Prov., Baoji City, Mei County, Jinqu Town, 14.VIII.2015, Zhenxia Ma & Yan Peng; 14♀♀11♂♂, China, Shannxi Prov., Baoji City, Mei County, Qinghua Town, Jinjiazhuang Village, 15.VIII.2015, Zhenxia Ma & Yan Peng; 34♀♀3♂♂, China, Shannxi Prov., Baoji City, Mei County, Huaiya Town, 16.VIII.2015, Zhenxia Ma & Yan Peng; 4♀♀1♂♂, China, Shannxi Prov., Ankang City, Zhenping County, Zengjia Town, 10.VIII.2015, Zhenxia Ma & Yan Peng; 4♂♂, China, Chongqing Municipality, Chengkou County, Xianyi Village, Tingjing Li & Chunfa Chen; 1♀1♂, China, Guizhou Prov., Tongren City, Jiang kou County, Minxiao Town, 29.VI.2015, Tingjing Li & Yan Peng; 1♀1♂, China, Guizhou Prov., Tongren City, Jiang kou County, Heiwan Villiage, 28.VI.2015, Tingjing Li & Yan Peng; 1♀22♂♂, China, Sichuan Prov., Dazhou City, Chengbei Town, Hongqi Village, 7.VI.2013, Ju You; 3♀♀49♂♂, China, Sichuan Prov., Dazhou City, Chengbei Town, Qingfeng Village, 4.VI.2013, Ju You; 7♀♀19♂♂, China, Sichuan Prov., Dazhou City, Maanshan, 10.VI.2013, Ju You; 3♀♀11♂♂, China, Sichuan Prov., Dazhou City, 8.VI.2013, Ju You; 17♂♂, China, Sichuan Prov., Chengdu City, Dujiangyan, Daguan Town, 16.VIII.2011, Tingjing Li & Yuan Bai; 3♀♀, China, Sichuan Prov., Leshan City, Emeishan County, Gaoqiao Town, Zhanggou Village, 8.VIII.2012, Tingjing Li; 3♀♀2♂♂, China, Sichuan Prov., Chongzhou City, Tianshun Village, 17.VIII.2011, Tingjing Li; 4♀♀8♂♂, China, Jiangxi Prov., Fuzhou City, Lichuan County, Hufang Villiage, 21.VI.2104, Tingjing Li.

#### Diagnosis.

Body strongly sculptured, with large and strong punctures, and extensive spots (Figs 69–72). Cephalic fovea almost obsolete; anterior surface of pronotum vertical, median foveae shallow and slightly U-shaped, a few long parallel transverse carinae above median foveae, pronotal carina complete (Fig. 69); propodeal shelf weak (Fig. 70); metasomal tergum II without a distinct apical lamella (Fig. 71); median longitudinal furrow on metasomal sternum II basally weak, even obsolete, and its anterior surface sloping (Fig. 72).

#### Distribution.

China (Sichuan, Jilin, Shaanxi, Chongqing, Guizhou, Jiangxi), Russia, Korea, Japan.

### Stenodynerus funebris

Taxon classificationAnimaliaHymenopteraVespidae

(André, 1884)

Figs 73–76

Odynerus funebris André, 1884: 729; Blüthgen 1955: 412.
Odynerus limbonatatus : Kostylev, 1940: 28.
Stenodynerus funebris : van der Vecht and Fischer 1972: 67; Gusenleitner 1981: 219, 243; Kim 1999: 347, 349, 350; Kim and Yamane 2004: 239, 255.

#### Material examined.

1♀1♂, China, Jilin Prov., Yanji City, Xiaoying Town, Minzhu Village, 3.VII.2012, Xin Zhou & Ju You.

#### Diagnosis.

Body obviously black (Fig. 73), only with the following spots yellow: mandibular basally, scape ventrally, inter-antennal spot, a minute post-ocular spot, pronotum sometimes with a pair of small spots, apical bands on metasomal terga I–II and sternum II, a long band on fore tibiae dorsally; cephalic fovea small and shallow, width less than the distance between posterior ocelli; clypeus medically convex, apex moderately emarginated (Fig. 74); anterior surface of pronotum almost vertical, with V-shaped median foveae, pronotal carina complete (Fig. 75); propodeal shelf developed, posterior surface concave, with a very short median longitudinal carina in lower portion (Fig. 76); median longitudinal furrow on metasomal sternum II basally weak, even obsolete, and its anterior surface sloping.

#### Distribution.

China (Shanxi, Jilin), Korea, Russia.

### Stenodynerus incurvitus

Taxon classificationAnimaliaHymenopteraVespidae

Gusenleitner, 2003

Stenodynerus incurvitus Gusenleitner, 2003: 855.

#### Material examined.

No specimens examined.

#### Diagnosis.

Clypeus yellow basally, with short setae, its width equal to its length; cephalic fovea comparatively larger, width more than the distance between posterior ocelli; propodeal concavity almost smooth; metasomal tergum II without a distinct apical lamella, punctures on apex deep; sternum II with a median longitudinal furrow basally, and its anterior surface sloping (Gusenleitner 2003).

#### Distribution.

China (Taiwan).

### Stenodynerus morawitzi

Taxon classificationAnimaliaHymenopteraVespidae

Kurzenko, 1977

Stenodynerus morawitzi Kurzenko, 1977: 554; Gusenleitner 1981: 219, 266.

#### Material examined.

No specimens examined.

#### Diagnosis.

Clypeus yellow, with small punctures, its width somewhat more than length, apex deeply emarginated; prontoal carina interrupted medially; tergum II with a broad reflex lamella apically. In male, metasomal terga I–VII and sterna II–III with yellow apical bands (Gusenleitner 1981).

#### Distribution.

China (Inner Mongolia).

### Stenodynerus morbillosus

Taxon classificationAnimaliaHymenopteraVespidae

Giordani Soika, 1979

Stenodynerus morbillosus Giordani Soika, 1979: 250; Gusenleitner 1981: 220, 286.

#### Material examined.

No specimens examined.

#### Diagnosis.

Larger species, body length generally more than 10 mm. Clypeus yellow; pronotal carina less developed; metasomal tergum II without a distinct apical lamella; sternum II with a short median longitudinal furrow basally (Giordani Soika 1979; Gusenleitner 1981).

#### Distribution.

China (northeast).

### Stenodynerus nudus

Taxon classificationAnimaliaHymenopteraVespidae

(Morawitz, 1889)

Lionotus nudus Morawitz, 1889: 164; Morawitz 1895: 463; von Schulthess 1934: 91.
Nannodynerus nudus : Blüthgen 1942: 320.
Stenodynerus nudus : van der Vecht and Fischer 1972: 68; Gusenleitner 1981: 219, 271.

#### Material examined.

No specimens examined.

#### Diagnosis.

Clypeus yellow except margin, with sparse and shallow punctures, its width much more than its length; metasomal tergum II without a distinct apical lamella; anterior surface of metasomal sternum II vertical. Metasomal terga I–V and sterna II–III with yellow apical bands (Gusenleitner 1981).

#### Distribution.

China (Inner Mongolia), Turkmenistan, Kazakhstan, Mongolia.

### Stenodynerus pappi luteifasciatus

Taxon classificationAnimaliaHymenopteraVespidae

Kim & Yamane, 2004

Stenodynerus pappi luteifasciatus Kim & Yamane, 2004: 235, 238, 245.

#### Material examined.

No specimens examined.

#### Diagnosis.

The species differs from *Stenodynerus pappi pappi* as follows: body with extensive orange-yellow spots; a broad band on pronotal dorsum and almost entire metanotum orange-yellow; punctures on anterior surface of pronotum sparser, apical band on tergum I broader and reflex apical lamella of metasomal tergum II narrower than the corresponding parts in *Stenodynerus pappi pappi* (Kim and Yamane 2004).

#### Distribution.

China (Taiwan).

### Stenodynerus pappi pappi

Taxon classificationAnimaliaHymenopteraVespidae

Giordani Soika, 1976

Figs 77–82

Stenodynerus pappi Giordani Soika, 1976: 290–291; Gusenleitner 1981: 219, 298; Kim 1999: 204; Kim and Yamane 2004: 237, 242.
Parancistrocerus ussuriensis Kurzenko, 1982 (1981): 117–122.

#### Material examined.

2♀♀, China, Shaanxi prov., Baoji City, Taibai County, Taochuan town, 12.VIII.2015, Zhenxia Ma & Yan Peng; 1♀, China, Shaanxi prov., Yanan City, Huanglong County, Shibao Town, 2.VIII.2012, Xin Zhou; 1♀, China, Shaanxi prov., Weinan City, Hua County, Jindui Town, 7.VIII.2012, Ju You & Yuan Bai; 1♀, China, Chongqing, Shizhu County, Huangshui Town, 12.VIII.2008, Bin Chen & Tingjing Li.

#### Diagnosis.

Cephalic fovea small and shallow, width less than the distance between posterior ocelli; clypeus black, sparsely punctate, with sparse setae, its width more than length (Fig. 77); anterior surface of pronotum sloping, with distinct punctures and a pair of round contiguous median foveae, the interspace between these two median foveae less than one diameter, pronotal carina obsolete (Fig. 80); propodeal shelf developed, posterior surface concave, with a long median longitudinal carina (Fig. 81); anterior surface of metasomal tergum I vertical, almost impunctate, and with a median longitudinal carina in upper half (Fig. 78); metasomal tergum II with a broad reflex lamella apically, dense and deep punctures forming a wide transverse groove on the base of lamella (Fig. 82); sternum II with a short median longitudinal furrow basally, and its anterior surface sloping (Fig. 79).

#### Distribution.

China (Zhejiang, Jiangxi, Shaanxi, Chongqing, Taiwan), Korea.

### Stenodynerus taiwanus

Taxon classificationAnimaliaHymenopteraVespidae

Kim & Yamane, 2004

Stenodynerus taiwanus Kim & Yamane, 2004: 235, 237, 241.

#### Material examined.

No specimens examined.

#### Diagnosis.

Anterior surface of pronotum distinctly punctate, with a pair of round contiguous median foveae, pronotal carina weak; propodeal shelf absent, posterior surface concave, with a median longitudinal carina lost in upper half; anterior surface of metasomal tergum I almost impunctate, dorsal surface with weak and sparse punctures, the interspaces between punctures more than one diameter. Whole body with sparse and long setae (Kim and Yamane, 2004).

#### Distribution.

China (Taiwan).

### Key to the Chinese species of the genus *Stenodynerus*

The characters are applicable to both sexes unless the sex is specified.

**Table:** 

1	Metasomal tergum II with a lamella apically (Figs 13, 29, 36, 61, 82)	**2**
–	Metasomal tergum II without a lamella apically (Figs 5, 44, 45, 50, 55, 59, 67, 71)	**8**
2	Anterior surface of metasomal tergum I vertical, with few punctures and a median longitudinal carina in upper half (Figs 10, 26, 33, 78)	**3**
–	Anterior surface of metasomal tergum I with strong punctures, not vertical and somewhat rounded, without a median longitudinal carina	**7**
3	Metasomal tergum II with a broad reflex lamella apically, the transverse groove on the base of lamella wider (Figs 13, 29, 82)	**4**
–	Metasomal tergum II with a narrow reflex lamella apically, the transverse groove on the base of lamella narrower (Fig. 36)	***Stenodynerus tenuilamellatus* sp. n.**
4	In female, clypeus black (Fig. 77); in profile, anterior surface of metasomal sternum II sloping (Fig. 79)	***Stenodynerus pappi*, 5**
–	In female, clypeus with yellow or ferruginous spots (Figs 8, 24); in profile, anterior surface of metasomal sternum II vertical (Figs 12, 28)	**6**
5	Body with yellow spots; pronotum with a pair of small spots dorsally (Fig. 80), metanotum with a yellow band anteriorly (Fig. 81), tergum I with a narrower apical band (Fig. 78)	***Stenodynerus pappi pappi* Giordani Soika**
–	Body with orange yellow spots; pronotal dorsum with a broad band, metanotum almost entire orange yellow, tergum I with a broader apical band (Kim and Yamane2004)	***Stenodynerus pappi luteifasciatus* Kim & Yamane**
6	Anterior surface of pronotum with distinct punctures, the interspace between median foveae less than one fovea diameter (Fig. 9); propodeal shelf developed (Fig. 11); anterior vertical surface of tergum I without a transverse striation (Fig. 10); sternum II with a very short median longitudinal furrow basally (Fig. 12)	***Stenodynerus reflexus* sp. n.**
–	Anterior surface of pronotum with few punctures, the interspace between median foveae more than one fovea diameter (Fig. 25); propodeal shelf weak (Fig. 27); anterior vertical surface of tergum I with two transverse striations (Fig. 26); sternum II without a median longitudinal furrow basally (Fig. 28)	***Stenodynerus strigatus* sp. n.**
7	In female, clypeus yellow; in male, metasomal terga I–VII and sterna II–III with yellow apical bands (Giordani Soika 1981)	***Stenodynerus morawitzi* Kurzenko**
–	In female, clypeus black; in male, metasomal terga I–II and sternum II with yellow apical bands	***Stenodynerus tergitus* Kim (new record)**
8	Larger species, body length generally more than 10 mm (Giordani Soika 1979; Giordani Soika 1981)	***Stenodynerus morbillosus* Giordani Soika**
–	Smaller species, body length generally less than 10 mm	**9**
9	Apical bands of metasomal terga I–V yellow or orange-yellow	**10**
–	Apical bands of metasomal terga I–II yellow or ferruginous	**11**
10	In profile, anterior surface of metasomal sternum II sloping (Fig. 53)	***Stenodynerus nepalensis* Giordani Soika (new record)**
–	In profile, anterior surface of metasomal sternum II vertical (Gusenleitner 1981)	***Stenodynerus nudus* (Morawitz)**
11	Propodeal concavity almost smooth (Gusenleitner 2003)	***Stenodynerus incurvitus* Gusenleitner**
–	Propodeal concavity with long and transverse rugae	12
12	Anterior surface of pronotum with a pair of round contiguous median foveae; anterior surface of metasomal tergum I almost impunctate (Kim and Yamane 2004)	***Stenodynerus taiwanus* Kim & Yamane**
–	Pronotal median foveae contiguous and forming U-shaped or V-shaped; anterior surface of metasomal tergum I with strong punctures (Figs 5, 59, 71)	**13**
13	Pronotal median foveae shallow and slightly U-shaped (Fig. 69)	***Stenodynerus frauenfeldi* (de Saussure)**
–	Pronotal median foveae distinct and V-shaped (Figs 3, 18, 40, 48, 57, 65, 75)	**14**
14	Clypeus with yellow or ferruginous spots; metasomal sternum II with a long median longitudinal furrow basally	**15**
–	Clypeus black; metasomal sternum II with a very short median longitudinal furrow basally, sometimes obsolete	**18**
15	Body with large and dense punctures, strongly sculptured (Fig. 63); anterior surface of pronotum sloping, and with distinct and strong punctures (Fig. 65)	***Stenodynerus chinensis chinensis* (de Saussure, 1863)**
–	Body with comparatively smaller punctures, moderately sculptured; anterior surface of pronotum almost vertical, with few punctures (Figs 3, 18, 40)	**16**
16	Clypeus strongly punctate (Fig. 2); apex of ocular sinus pale ferruginous; pronotal carina complete	***Stenodynerus ningliangensis* sp. n.**
–	Clypeus moderately punctate (Figs 16, 39); apex of ocular sinus black; pronotal carina interrupted medially	**17**
17	Anterior surface of pronotum with wider V-shaped median foveae (Fig. 18); apex of metasomal tergum II with somewhat shallower and sparser punctures (Fig. 45); in male, volsella slightly truncate apically (Fig. 20)	***Stenodynerus similibaronii* sp. n.**
–	Anterior surface of pronotum with narrower V-shaped median foveae (Fig. 40); apex of metasomal tergum II with strongly deep and dense punctures (Fig. 44); in male, volsella with rounded apically (Figs 38, 41)	***Stenodynerus baronii* Giordani Soika (new record)**
18	Body with pale yellow spots; metasomal sternum II black except lateral surface (Fig. 49); metasomal tergum II slightly punctate (Fig. 50); sternum II without a median longitudinal furrow basally (Fig. 49)	***Stenodynerus bluethgeni* van der Vecht (new record)**
–	Body with yellow spots; metasomal sternum II with a narrow apical band (Fig. 58); metasomal tergum II strongly punctate (Figs 59, 73); sternum II with a very short median longitudinal furrow basally (Fig. 58)	**19**
19	Anterior surface with a few short transverse carinae above median foveae (Fig. 75); propodeal shelf developed (Fig. 76)	***Stenodynerus funebris* (André)**
–	Anterior surface without a transverse carina above median foveae (Fig. 57); propodeal shelf obsolete	***Stenodynerus pullus* Gusenleitner (new record)**

## Supplementary Material

XML Treatment for Stenodynerus

XML Treatment for Stenodynerus ninglangensis

XML Treatment for Stenodynerus reflexus

XML Treatment for Stenodynerus similibaronii

XML Treatment for Stenodynerus strigatus

XML Treatment for Stenodynerus tenuilamellatus

XML Treatment for Stenodynerus baronii

XML Treatment for Stenodynerus bluethgeni

XML Treatment for Stenodynerus nepalensis

XML Treatment for Stenodynerus pullus

XML Treatment for Stenodynerus tergitus

XML Treatment for Stenodynerus chinensis chinensis

XML Treatment for Stenodynerus copiosus

XML Treatment for Stenodynerus frauenfeldi

XML Treatment for Stenodynerus funebris

XML Treatment for Stenodynerus incurvitus

XML Treatment for Stenodynerus morawitzi

XML Treatment for Stenodynerus morbillosus

XML Treatment for Stenodynerus nudus

XML Treatment for Stenodynerus pappi luteifasciatus

XML Treatment for Stenodynerus pappi pappi

XML Treatment for Stenodynerus taiwanus
